# Poor Bone Quality in Patients With Amyotrophic Lateral Sclerosis

**DOI:** 10.3389/fneur.2020.599216

**Published:** 2020-12-18

**Authors:** Jordi Caplliure-Llopis, Dolores Escrivá, María Benlloch, José Enrique de la Rubia Ortí, José María Estrela, Carlos Barrios

**Affiliations:** ^1^School of Doctorate, Valencia Catholic University, Valencia, Spain; ^2^Primary Care Services, La Ribera University Hospital, Alzira, Spain; ^3^Intensive Care Unit, La Fe Polytechnic and University Hospital, Valencia, Spain; ^4^Department of Anatomy and Physiology, School of Medicine and Health Sciences, Valencia Catholic University, Valencia, Spain; ^5^Department of Physiology, University of Valencia, Valencia, Spain; ^6^Institute of Research on Musculoskeletal Disorders, Valencia Catholic University, Valencia, Spain

**Keywords:** amyotrophic lateral sclerosis, bone quality, neurodegeneration, osteoporosis, quantitative ultrasound (QUS) measurement, vitamin D

## Abstract

**Objective:** Musculoskeletal functional deterioration in Amyotrophic lateral sclerosis (ALS) is associated with an increase in bone fractures. The purpose of this study was to evaluate the influence of sex, ALS type, on bone quality in patients with ALS compared to healthy controls. The impact on bone health of the clinical status and some metabolic parameters was also analyzed in ALS patients.

**Methods:** A series of 33 voluntary patients with ALS, and 66 healthy individuals matched in sex and age underwent assessment of bone mass quality using quantitative ultrasound (QUS) of the calcaneus. Ultrasonic broadband attenuation (BUA), the speed of sound (SOS), stiffness index and *T*-score were measured. Bone mineral density (BMD) was estimated using standard equations. Apart from fat and muscle mass percentage determinations, clinical baseline measures in ALS patients included ALSFRS-R score, Barthel index for activities of daily living, pulmonary function measured using FVC, and muscular strength assessed by a modified MRC grading scale. Laboratory tests included serum calcium, 25-HO-cholecalciferol (Vitamin D), alkaline phosphatase (ALP), T4 and TSH.

**Results:** All bone parameters evaluated were statistically significant lower in ALS patients than in healthy controls. ALS females showed significantly lower bone parameters than healthy females. According to the estimated BMD, there were 25 ALS patients (75.8%) and 36 (54.5%) healthy individuals showing an osteoporotic profile (BMD <0.700 g/cm^2^). Only 16.7% of the ALS females had *T*-scores indicative of healthy bones. There was no correlation between any of the clinical parameters analyzed and the bone QUS measurements. Vitamin D and TSH levels positively correlated with all the bone parameters.

**Conclusions:** This study confirms that ALS patients, particularly females, exhibited deteriorated bone health as compared to healthy individuals. These structural bone changes were independent of ALS subtype and clinical status. Bone health in ALS patients seems to be related to certain metabolic parameters such as Vitamin D and TSH levels.

## Introduction

Amyotrophic lateral sclerosis (ALS) is a devasting neurodegenerative disease with progressive motor neuron damage leading to the death of the patients in a very short average time, from 16 months to 3 years after diagnosis (1). Epidemiologically, ALS is considered a rare disease with an annual incidence of 2–3 cases per 100,000 people (1). There are many studies that show an increased risk in males (male/female ratio of 1.5), indicating that a hormonal component could be involved. However, as age increases, the difference between men and women disappears (2–4).

From a clinical point of view, two main types of ALS are distinguished: bulbar and medullary, depending on whether the upper or lower motor neuron, respectively, is initially affected. In both cases, a series of characteristic signs and symptoms appear, reflecting progressive musculoskeletal deterioration. Muscular dysfunction at the thoracic level and subsequent respiratory failure usually are the final cause of death (5). Musculoskeletal functional deterioration in ALS is associated with an increase in bone fractures, due to a decrease in bone density (6–8).

Bone is an active organ in constant remodeling and direct interactions with the skeletal muscle. These muscles types provide certain anabolic stimuli for bone remodeling, and are source of osteogenic stem cells that play an important role in maintaining bone homeostasis (9, 10). In the opposite sense, bone is an important supplier of muscular trophic factors, including VEGF, FGF-2, BMP, and IGF-1 that are critical for the maintenance of muscle homeostasis. Furthermore, the shape of the bones is constantly regulated by muscle strength, so when muscle dysfunction occurs, such as in the early stages of ALS, bone morphology can be altered (11). Similarly, the alteration of bone homeostasis could exacerbate muscle degeneration and accelerate the progression of the disease. By contrast, the preserved paracrine release of VEGF by the mesenchymal stem cells (MSCs) of the bone marrow may improve muscle regeneration (11). In this sense, the MSCs of patients with ALS produce fewer trophic factors (12, 13). The results of these studies suggest a possible interaction between muscle and bone during the progression of ALS disease.

Based on the previous findings, the objective of this study was to analyze the bone quality of patients with ALS in relation to sex, ALS type, and age.

## Materials and Methods

### Participants

This is cross-sectional descriptive study of bone quality on patients with ALS disease that were included in the list to initiate a single-center, randomized, double-blinded, registered clinical trial (ClinicalTrials.gov, NCT03489200). Participants fulfilled the following inclusion criteria: age over 18-years-old, diagnosed with ALS according to the criteria of El Escorial (14) (sporadic or familial), with symptoms persisting more than 6 months, and both sexes. The clinical subtype of ALS, bulbar or spinal, was taken from the clinical records. Those females who were breastfeeding or pregnant at the time of the study were excluded. At the time of evaluation, all patients were taking Riluzole® according to the standard dose prescribed by their neurologist. After applying the selection criteria, a total of 33 patients participated in the study. The patients were affiliated to the Spanish Foundation for Research on ALS (FUNDELA). All participants provided their written and voluntary consent.

A control group of 66 healthy individuals within the same range of age and similar sex distribution than the group of ALS patients was recruited to participate in the study. The ALS/healthy controls ratio was 1:2 maintaining similar proportion of males and females in the control group than the incidence of the disease by sex observed in ALS group. Similar to patients with ALS, all then voluntarily agreed to be involved in the study, after the objectives of the project and procedures were explained to them.

The study was developed in accordance with the Declaration of Helsinki (15) after approval of the protocol by the Human Research Committee of the Commission for Ethics in Experimental Research of the University of Valencia (reference number H1479983999044).

### Anthropometric, Clinical and Metabolic Measurements

The weight and height of each participant were measured and recorded using the same standardized equipment under regular calibration. The body mass index (BMI) was calculated with the weight (kg) divided by the square of the height in meters (m^2^). In ALS patients, fat and muscle mass percentage were obtained according to standard procedures (16). The main clinical baseline measures included the ALSFRS-R score (17), Barthel index for activities of daily living, pulmonary function measured using FVC, and muscular strength assessed by a modified 11-step MRC grading scale (18). MRC scoring was obtained in each patient for 8 different muscle: right and left biceps, triceps, quadriceps and tibial. To calculate the total MRC scale index, a progressively increasing number from 0 (0 in the MRC scale) to 10 (5 in the MRC scale) was given to each step and each muscle. The total MRC index per patient corresponds to the sum of the numbers given to all the eight muscles. Laboratory tests included serum calcium, 25-HO-cholecalciferol (Vitamin D), alkaline phosphatase (ALP), T4 and TSH.

### Assessment of Bone Quality

Quantitative ultrasound measurement (QUS) was used for the evaluation of bone mass parameters using the Lunar Achilles Insight device (GE Healthcare, Little Chalfont, United Kingdom). This technique was chosen for its ability to estimate bone quality in a short time, ease of application and reproducibility and the absence of adverse effects.

The Lunar Achilles Insight device is a portable device that allows a quick estimate of two basic parameters: the ultrasonic broadband attenuation (BUA) and the speed of sound (SOS). BUA refers to the absorption of energy by bone and soft tissue when sound waves travel through them; an increase in BUA correlates with increased bone trabecula content and the unit is dB/MHz. The SOS parameter refers to the ratio of the length of the body part studied to the transmission time of the sound waves. Its increase is correlated with reduced bone mineral content. The unit of measurement is meters per second (m/s) (19). Both ultrasound parameters constitute a clinical variable called stiffness index (SI). The SI is expressed as a percentage of the mean value in a young adult and is calculated as follows: Stiffness index = 0.67 × (BUA) = 0.28 × (SOS) – 420. This parameter should not be confused with the biomechanical term. The SI has been used to determine the risk of osteoporotic fractures as has been shown to produce better effective precision than BUA or SOS alone and is also comparable to bone mineral density measured by the DXA method (20, 21).

Two ultrasound evaluations of the calcaneus of each subject under study were carried out and the mean of both values was later calculated. All ultrasound measurements were performed by the same operator, thus avoiding biases in obtaining the data. To obtain the bone mass, the instructions specified by the manufacturer were followed, spraying the calcaneus area with 70° alcohol and correct foot placement. The measurements were always made on the dominant foot. In our laboratory, the intra-operator variability for BUA and SOS was 13.7 and 3.2%, respectively. In accordance with the WHO criteria for BUA, *T*-scores equal to or <-1.5 were considered an indication of osteoporosis (22). The estimated heel BMD was calculated using the equation BMD = 33 × (BUA + SOS) – 3.687 (23). Data were collected at clinical facilities dependent on the Catholic University San Vicente Martir (Valencia, Spain).

### Statistical Analysis

Statistical analysis was performed with the SPSS version 21 software package (IBM Corporation, Chicago, IL, USA). Quantitative values were expressed as mean, standard deviation (SD), and 95% confidence intervals. The Gaussian distribution of the variables was evaluated using the Shapiro-Wilk test. Due to the limited sample size, comparisons between groups were made by using the Mann-Whitney U non-parametric test. A sub-analysis of the sample regarding sex and ALS subtype, bulbar or spinal, was also performed. Spearman's rho correlation coefficients were calculated to assess the possible relationship between bone quality parameters and anthropometric variables. *P* < 0.05 were considered statistically significant.

## Results

The total population that was finally included in the study was 99 individuals (33 ALS patients and 66 healthy controls). Age and the general anthropometric characteristics of the series are shown in Table 1 including date discriminated by sex. The proportion of males was similar (63.6%) in both ALS and healthy controls series. Considering the mix sample of males and females, there were statistically significant differences either in weight (*p* < 0.01) and BMI (*p* < 0.05) between the two groups. Both males and females with ALS showed lower weight and BMI than healthy participants. Differences were only statistically significant comparing males of both groups (*p* < 0.01 for weight, and p < 0.05 for BMI). Healthy males were slightly younger than ALS males, and healthy females slightly older than ALS counterparts, but there were no statistically significant differences.

**Table 1:** Age and general anthropometric characteristics of healthy controls and ALS patients included in the study and discriminated by sex.

	**Healthy controls Mean** **±** **SD (95% CI)**			**ALS Mean** **±** **SD (95% CI)**			**Healthy vs. ALS Z (*****p*****) Mann-Whitney** ***U*****-test**		
	**Total sample (*n* = 66)**	**Males (*n* = 42)**	**Females (*n* = 24)**	**Total sample (*n* = 33)**	**Males (*n* = 21)**	**Females (*n* = 12)**	**Total sample**	**Males**	**Females**
Age (years)	54.6 ± 8.1 (52.6–56.7)	53.6 ± 7.2 (51.4–55.8)	56.5 ± 9.5 (52.5–60.5)	55.6 ± 10.6 (51.7–59.4)	56.3 ± 10.8 (51.2–61.4)	54.2 ± 10.4 (47.6–60.8)	−0.008 (0.994)	−0.482 (0.630)	−1.025 (0.305)
Weight (Kg)	75.8 ± 12.6 (72.7–78.9)	82.4 ± 9.3 (79.5–85.3)	64.4 ± 8.9 (60.6–68.2)	69.0 ± 9.1 (65.7–72.2)	73.4 ± 7.6 (69.9–76.8)	61.2 ± 5.9 (57.4–65.0)	−2.629 (0.009)**	−3.401 (0.001)**	−0.672 (0.501)
Height (cm)	170.4 ± 8.4 (168.3–172.5)	174.8 ± 6.2 (172.9–176.8)	162.6 ± 5.5 (160.3–165.0)	168.3 ± 8.5 (165.3–171.4)	172.0 ± 7.1 (168.7–175.3)	162.3 ± 7.4 (157.6–167.1)	−1.198 (0.263)	−1.569 (0.117)	−0.286 (0.775)
BMI (kg/m^2^)	26.0 ± 3.2 (25.2–26.8)	26.9 ± 2.7 (26.1–27.8)	24.3 ± 3.4 (22.9–25.8)	24.4 ± 2.7 (23.4–25.4)	25.1 ± 2.5 (23.9–26.2)	23.3 ± 2.8 (21.5–25.1)	−2.201 (0.028)*	−0.520 (0.021)*	−0.523 (0.603)

* *p < 0.05*;

** *p < 0.01*.

### Clinical Characteristics of ALS Patients

The clinical profile of the ALS patients included in the study are summarized in Table 2. Discrimination of data according to sex was also indicated. Regarding the type of ALS, 22 patients debuted with spinal disease and 11 with primary bulbar symptoms. No differences were found in the distribution of ALS clinical type by sex (Fisher's exact test; *p* = 0.471). The mean duration of symptoms was 2 years and the shorter period was 6 months. When discriminating by sex, the duration of symptoms was longer in males than in females, without statistically significant differences. Females with ALS showed slightly ALSFRS-R scores and slightly lower Barthel index than males, but without statistically significant differences. These data indicated that the clinical status of males and females with ALS was almost similar. There were no differences either in FEV% and muscle%. However, ALS females showed higher fat% than males (*p* < 0.05). Regarding laboratory parameters, Ca, ALP, and TSH values were quite similar, both within the range of normality. However, mean values of Vitamin D were in the low range of normality, with 44.4% of patients showing clear Vitamin D levels below the normality (<20 ng/l). Surprisingly, T4 values were lower in males than in females (*p* < 0.05) besides no differences in TSH values.

**Table 2:** Clinical profile and laboratory parameters of ALS patients.

	**ALS patients Mean** **±** **SD (95% CI)**			**Males vs. FemalesMann-Whitney *U*-test**
	**Total sample**	**Males**	**Females**	**Z (*****p*****)**
	**(*****n*** **=** **33)**	**(*****n*** **=** **21)**	**(*****n*** **=** **12)**	
Clinical profile				
Spinal/bulbar	22/11	15/6	7/5	
Duration of symptoms (months)	24.1 ± 17.8 (17.5–30.6)	28.1 ± 19.6 (18.9–37.3)	16.9 ± 11.4 (9.1–24.5)	−1.514 (0.130)
ALSFRS-R score	34.9 ± 8.8 (31.3–38.4)	34.4 ± 10.1 (20.3–39.4)	36.1 ± 5.2 (31.7–40.5)	−0.056 (0.978)
Barthel index	75.3 ± 22.9 (63.8–86.7)	79.1 ± 24.0 (63.9–94.4)	67.5 ± 20.4 (46.0–88.9)	−1.100 (0.271)
FEV%	77.0 ± 13.9 (68.9–85.0)	80.3 ± 13.2 (70.8–89.7)	68.7 ± 13.5 (47.4–90.3)	−1.346 (0.178)
Fat%	18.2 ± 3.8 (16.4–19.9)	16.9 ± 2.8 (15.4–18.5)	21.1 ± 4.8 (16.1–26.1)	−2.258 (0.024)*
Muscle%	36.8 ± 3.1 (32.2–40.6)	37.0 ± 2.8 (35.4–38.3)	36.4 ± 3.9 (32.2–40.6)	−0.467 (0.640)
MRC global score	58.2 ± 16.4 (50.7–65.7)	59.6 ± 17.0 (49.7–69.3)	55.6 ± 16.0 (40.7–70.3)	−0.674 (0.500)
Laboratory parameters				
25-OH-Vit D (ng/mL)	21.8 ± 7.8 (15.8–27.8)	22.5 ± 7.3 (15.6–29.3)	19.5 ± 12.1 (9.1–28.2)	−0.586 (0.558)
Calcium (mg/dL)	9.4 ± 0.3 (9.2–9.6)	9.5 ± 0.2 (9.3–9.6)	9.3 ± 0.5 (8.5–10.1)	−0.568 (0.570)
Alkaline phosphatase UI/L	64.3 ± 21.1 (54.4–74.1)	65.1 ± 22.3 (52.7–77.5)	61.8 ± 18.7 (38.5–85.1)	−0.087 0.930
T4 (ng/dL)	1.9 ± 2.3 (0.45–3.4)	1.1 ± 0.2 (0.9–1.2)	3.3 ± 3.60 (−2.4–9.1)	−2.457 (0.014)*
TSH (uU/mL)	1.6 ± 0.6 (1.3–1.9)	1.7 ± 0.5 (1.3–1.9)	1.5 ± 0.8 (0.8–2.3)	−0.463 (0.663)

* *p < 0.05*.

### Bone Parameters

Table 3 shows the results of the QUS evaluation and the estimated BMD in the two groups, including data discriminated by sex. There were differences between the total samples of both groups in all the bone parameters analyzed. Except in healthy control males, the mean estimated BMD were below the values considered as indicator of osteoporosis (<0.700 g/cm^2^) but were lower in ALS patients (*p* = 0.012). There were 25 ALS patients (75.8%) who BMD values indicative of osteoporosis and 36 (54.5%) healthy individuals (Fisher's exact test, *p* < 0.05).

**Table 3:** Bone parameters obtained by QUS in the two groups of participants.

	**Healthy controls Mean** **±** **SD (95% CI)**			**ALS Mean** **±** **SD (95% CI)**			**Healthy vs ALS Mann-Whitney** ***U***-test Z* **(*****p*****)**		
	**Total (*n* = 66)**	**Males (*n* = 42)**	**Females (*n* = 24)**	**Total (*n* = 33)**	**Males (*n* = 21)**	**Females (*n* = 12)**	**Total**	**Males**	**Females**
BUA (dB/MHz)	128.0 ± 12.7 (124.9–131.2)	131.6 ± 12.9 (127.5–135.6)	121.8 ± 9.9 (117.6–126.0)	113.9 ± 22.5 (105.9–121.9)	121.2 ± 21.5 (111.4–130.9)	101.2 ± 19.2 (89.1–113.5)	−3.125 (0.002)**	−1.823 (0.068)	−3.290 (0.001)**
SOS (m/s)	1560.2 ± 42.1 (1549.8–1570.6)	1566.7 ± 47.5 (1551.9–1581.5)	1548.8 ± 28.0 (1537.0–1560.7)	1539.7 ± 59.5 (1518.6–1560.8)	1559.3 ± 60.2 (1531.9–1586.8)	1505.3 ± 40.8 (1479.3–1531.3)	−2.316 (0.021)*	−0.707 (0.479)	−3.121 (0.002)**
I–stiffness	101.9 ± 18.7 (97.3–106.5)	105.9 ± 20.3 (99.6–112.3)	94.8 ± 13.2 (89.2–100.4)	86.8 ± 30.8 (75.9–97.8)	97.1 ± 30.1 (83.3–110.8)	68.9 ± 23.8 (53.7–84.0)	−2.754 (0.006)**	−1.269 (0.205)	−3.323 (0.001)**
*T*–score	0.3 ± 1.2 (0.0–0.6)	0.4 ± 1.4 (−0.1–0.8)	0.3 ± 0.9 (−0.1–0.6)	−1.01 ± 2.3 (−1.8 to −0.1)	−0.2 ± 2.3 (−1.2–0.8)	−2.3 ± 1.8 (−3.5 to −1.2)	−3.311 (0.001)**	−1.232 (0.218)	−3.897 (0.000)**
BMD (g/cm^2^)	0.689 ± 0.136 (0.664–0.722)	0.715 ± 0.151 (0.667–0.762)	0.643 ± 0.092 (0.604–0.682)	0.599 ± 0.209 (0.525–0.673)	0.668 ± 0.207 (0.574–0.763)	0.477 ± 0.153 (0.379–0.575)	−2.501 (0.012)*	−0.962 (0336)	−3.255 (0.001)**

* *p < 0.05*;

** *p < 0.01*.

When analyzing QUS bone parameters according to sex, no significant differences were surprisingly found in any of the parameters between males of the ALS group compared to those of the control group (Table 3). Slightly higher values were observed in all bone parameters of the healthy males, especially in BUA, *T*-scores and estimated BMD. However, when comparing females, those with ALS exhibited statistically significant lower values than healthy women in all the determined bone parameters. Among ALS females, values were much lower in BUA, SOS, SI, and *T*-score.

On the basis of the *T*-score alone, 51.5% (15/33) of the ALS patients showed values below −1.5, considered as indicators of osteopenia when using QUS examinations. In the control group only 4 individuals (6.1%) had a *T*-score of below 1.5 and all 4 were males. The differences between the ALS and control groups in the proportion of osteopenic individuals was highly significant (*p* < 0.0001). Only 16.7% of the ALS females (2/12) had *T*-scores indicative of healthy bones.

Concerning the BMD estimated values according to sex, no significant differences were obtained between the men in the control group and those in the ALS group. Among females, those with ALS exhibited much lower BMD values than healthy women (*p* = 0.001). Most of the ALS females were within the range of BMD considered as osteoporosis (11/12), but only 4 out of 24 healthy females were within the osteoporosis range (*p* < 0.0001). Among males, 7 out of 21 ALS patients had BMD values below 0.700 mg/cm^2^, and 23 out of 42 in the healthy group. Differences in the proportion of osteoporotic males were not statistically significant between the two groups (Fisher's exact test*, p* = 0.468).

QUS bone parameters did not depend on the of the disease type (bulbar or spinal ALS) (Table 4). Although spinal ALS showed impaired Barthel index (*p* < 0.05), higher fat % (*p* < 0.05), less muscle % (*p* < 0.01), and lower TSH levels (*p* < 0.05), no statistically significant differences were found in any of the bone parameters measured (Table 4).

**Table 4:** Clinical profile, laboratory tests and bone parameters according ALS subtype.

	**ALS Mean** **±** **SD (95% CI)**		
	**Bulbar (*N* = 11)**	**Spinal (*N* = 22)**	**Mann-Whitney Z (*p*)**
Clinical profile			
Time from diagnosis (months)	29.3 ± 20.1 (13.8–44.7)	22.0 ± 16.9 (14.5–29.4)	−1.071 (0.284)
ALSFRS-R score	36.7 ± 6.5 (31.7–41.7)	33.9 ± 9.9 (28.8–39.0)	−0.405 (0.685)
Barthel index	94.0 ± 13.4 (77.3–110.6)	68.1 ± 22.0 (54.7–81.3)	−2.265 (0.026)*
FEV%	74.4 ± 13.6 (61.7–87.0)	79.5 ± 14.7 (65.9–93.2)	−0.768 (0.442)
BMI	24.2 ± 2.4 (22.5–25.9)	24.7 ± 2.8 (23.4–25.9)	−0.569 (0.589)
Fat%	15.1 ± 3.2 (11.7–18.4)	19.4 ± 3.5 (17.4–21.3)	−2.180 (0.029)*
Muscle%	39.9 ± 2.2 (37.6–42.2)	35.6 ± 2.5 (34.2–37.1)	−2.725 (0.006)**
MRC global score	73.0 ± 7.1 (65.5–80.4)	52.3 ± 15.3 (43.8–60.8)	−2.618 (0.009)**
Laboratory test			
25-OH-VitD (ng/mL)	27.3 ± 6.8 (16.4–38.2)	17.4 ± 5.7 (10.4–24.5)	−1.715 (0.086)
Calcium (mg/dL)	9.5 ± 0.4 (9.0–9.9)	9.4 ± 0.2 (9.2–9.6)	−0.453 (0.662)
Alkaline phosphatase (UI/L)	57.5 ± 9.6 (47.4–67.5)	67.2 ± 24.1 (53.2–81.2)	−1.279 (0.201)
T4 (ng/dL)	1.2 ± 0.2 (0.8–1.6)	2.1 ± 2.6 (0.1–4.4)	−0.570 (0.575)
TSH (uU/mL)	2.1 ± 0.4 (1.6–2.5)	1.4 ± 0.6 (1.1–1.7)	−2.144 (0.032)*
Bone parameters			
I-stiffness	86.8 ± 33.7 (64.1–109.5)	86.9 ± 30.1 (73.5–100.2)	−0.076 (0.939)
BUA (dB/MHz)	115.4 ± 25.1 (98.5–132.4)	113.1 ± 21.7 (103.5–122.8)	−0.229 (0.819)
SOS (m/s)	1535.3 ± 63.2 (1492.9–1577.8)	1541.8 ± 58.9 (1515.3–1568.0)	−0.496 (0.620)
*T*-score	−1.0 ± 2.6 (−2.7–0.7)	−1.0 ± 2.3 (−2.0–0.0)	−0.115 (0.909)
BMD (g/cm^2^)	0.592 ± 0.226 (0.439–0.744)	0.602 ± 0.205 (0.511–0.693)	−0.191 (0.849)

* *p < 0.05*;

** *p < 0.01*.

### Correlation Between Clinical, Metabolic, and Bone Parameters

There was no correlation between any of the clinical parameters analyzed and the bone QUS measurements, indicating that ALS clinical profile has not a direct impact on bone health in this sample. However, two laboratory test, 25-OH-Vitamin D and TSH were related with bone quality parameters. Vitamin D strongly and positively correlated with BUA (Spearman's r: 0.867, *p* = 0.002), SOS (r: 0.750, *p* = 0.020), SI (r: 0.783, *p* = 0.013), *T*-score (r: 0.800, *p* = 0.010), and estimated DMO (r: 0.783, *p* = 0.013). TSH levels showed also a positive but less strong correlation with *T*-score (r: 0.470, *p* = 0.036), SI (r: 0.459, *p* = 0.042), and BUA (r: 0.451, *p* = 0.046). Serum calcium, ALP and T4 did not show correlation with any of the bone parameters.

## Discussion

In this cross-sectional study, bone quality parameters of patients with ALS measured by QUS were compared with those of a group of healthy individuals matched in age and anthropometric variables. There were differences in bone quality parameters between the ALS and the control groups. ALS participants showed lower bone quality (lower SOS, lower BUA, lower SI, lower *T*-score, and lower estimated BMD) than the control group. Notably, the differences in the bone quality variables were only statistically significant in females. The ALS group was stratified into 2 subtypes (bulbar/medullary) and no significant differences were found between the subgroups.

This study, therefore, confirms the hypothesis that ALS patients may exhibit deteriorated bone health with lower bone density than healthy individuals. These structural bone changes cannot be attributed to the aging process. Interestingly, the youngest ALS patients in this series showed BMD estimated values quite similar to those found in older ALS patients, showing a lack of correlation between age and BMD as found in healthy individuals. However, the poor bone health detected in this group of ALS patients could be a consequence of changes in diet, physical inactivity, lack of sun exposure, medication use, or other unknown features linked to the disease that were not addressed in the current research.

An increased bone turnover was recently detected in ALS patients after measurement of blood biomarkers (24). Furthermore, Vitamin D deficiency that could affect bone health including bone turnover and bone mineral density was observed in ALS patients (8, 25, 26). In the current study, neither bone turnover markers nor vitamin D metabolism were measured so the relationship between deterioration of bone quality parameters and biomarkers could not be explored.

Various case-controlled studies have described an association between ALS and fracture occurrence (27, 28). Some of these studies included a mix of ALS patients and cases with progressive muscular atrophy. Recently, a prospective study showed a positive association between ALS and fracture incidence, both osteoporotic and non-osteoporotic and of both traumatic and non-traumatic origin (29). The increased incidence of fractures in ALS patients suggests poor bone health as an underlying factor in several neurodegenerative diseases (8, 25, 26, 30). None of our ALS patients reported fractures before the start of the study. As most of them exhibited poor bone quality as assessed by QUS, a prospective study regarding the occurrence of fractures in the future could be worthwhile.

Most of the experimental research on ALS uses the SOD1G93A mouse model. Interestingly, a reduction in trabecular and cortical bone mass reducing the mechanical properties of bone was found in this ALS animal model (31, 32). The bone loss was related to decreased bone formation and enhanced bone resorption. In addition to the deteriorated skeletal muscle function, these findings reflect signs of skeletal fragility in ALS animal models as indicated also by reduced femoral bending properties and fracture resistance compared to control mice (32). This latter study concluded that the loss of bone mass was due to reduced bone formation and increased resorption. Similarly, analyzing the same ALS mice model, a reduced bone mass associated with multiple impairments of osteoblast properties and striking acceleration of osteoclast formation in bone was found only in those animals with severe muscle atrophy (31).

The results of the current study on ALS patients are in agreement with previous observations on ALS animal models and, therefore, may have important repercussions for clinical practice. ALS patients have poor bone quality mediating a skeletal fragility that is still not well-understood. In addition to a deeper knowledge of the underlying mechanisms of neurodegeneration, a possible muscle-bone crosstalk in ALS is currently gaining attention among investigators (12).

At present, there is increasing evidence about the lifelong interaction of skeletal muscles and bone remodeling (33–37). Skeletal muscles offer important anabolic stimuli for bone turnover through the delivery of osteogenic growth factors, such as fibroblast growth factor (FGF-2) and insulin-like growth factor (IGF-1) (38–41). Moreover, maintaining bone homeostasis is closely related to muscle-derived stem cells (42–44). Finally, from a morphologic developmental perspective, the muscle-induced mechanical loading of bones regulates not only the shape of the bones but also crucial aspects of bone morphogenesis (37, 40, 41, 45, 46). However, the basic molecular mechanisms behind the potential muscle-bone interactions still remain unclear.

ALS is characterized by a severe muscle atrophy that theoretically leads to gradual reduction of the mechanical loading to the skeleton and consequently to a disorder of bone remodeling and morphology that may be important as the disease progresses. Muscle paralysis in ALS patients rapidly affects the respiratory function leading to death. Therefore, bone health impairment in ALS patients and its possible involvement in disease progression have rarely been addressed in the literature. The few clinical studies on bone quality in ALS patients confirm that these patients exhibit decreased bone mineral density, elevated serum bone turnover markers and a higher fracture incidence (8, 47). The novelty of the current research is the influence of sex on bone structural properties in ALS patients, particularly in young adults. Women with ALS showed a greater bone loss than their healthy counterparts. A convincing explanation for this sex-linked bone deterioration not involving aging is unclear and deserves further research.

For clinicians, the prevention of skeletal fractures should be more extensively considered since fracture occurrence could severely impair the quality of life in longer-surviving ALS patients. Drugs inhibiting bone resorption such as bisphosphonates or Denosumab and drugs promoting bone formation such as teriparatide, should be included in the therapeutic arsenal of ALS patients. Supplementation with vitamin D should be also considered in ALS patients with Vitamin D deficiency since a delayed decline in the ALSFRS-R score was found in some studies (25, 26). In the current study, Vitamin D levels positively correlated to all the bone parameters analyzed, even with the estimated BMD. These results emphasized the monitoring of Vitamin D in ALS patients in terms of bone health preservation and fracture prevention.

This study has obviously some limitations. First, the cohort included prevalent cases with more than 6 months of symptoms that were included in a list to initiate a single-center RCT. The series did not include newly diagnosed cases. Our hospital is a reference center for ALS treatment; all new cases diagnosed in the community area are derived to our ALS unit. There are no other patients treated outside our Hospital in our health area. We honestly believe that this cohort of ALS patients represented a true population-based sample. A second limitation is related to the QUS technique that does not directly measure BMD. However, this non-invasive technique provides good estimators (BUA and SOS) of bone quality such as the microarchitecture, elasticity, and density. Furthermore, QUS is sensitive to differences in bone porosity and matrix properties between individuals (48). Independently of certain variability of data, BUA and SOS have been found valuable in predicting fracture risk (49).

In conclusion, this study confirms that ALS patients, particularly females, exhibit deteriorated bone health with lower bone density than healthy individuals. These structural bone changes are independent of the clinical status and ALS subtype, and cannot be attributed to the aging process. Bone health in ALS patients sems to be related to certain metabolic parameters such as Vitamin D and TSH levels. Further research is required to determine the full impact of neurodegenerative diseases such as ALS on bone metabolism.

## Data Availability Statement

The raw data supporting the conclusions of this article will be made available by the authors, without undue reservation.

## Ethics Statement

The studies involving human participants were reviewed and approved by Human Research Committee of the Commission for Ethics in Experimental Research of the University of Valencia (reference number H1479983999044). The patients/participants provided their written informed consent to participate in this study.

## Author Contributions

JC-L, JE, and CB designed the research. JC-L and DE recorded the data. JC-L, DE, MB, JR, JE, and CB analyzed the data, read, and approved the manuscript. All authors contributed to the writing of the manuscript. All authors contributed to the article and approved the submitted version.

## Conflict of Interest

The authors declare that the research was conducted in the absence of any commercial or financial relationships that could be construed as a potential conflict of interest.
